# Efficient nanostructured materials to reduce nutrient leaching to overcome environmental contaminants

**DOI:** 10.1038/s41598-024-54049-1

**Published:** 2024-02-27

**Authors:** Farwa Nadeem, Muhammad Asif Hanif, Najla AlMasoud, Taghrid S. Alomar, Adnan Younis

**Affiliations:** 1 Nano and Biomaterials Lab, Department of Chemistry, University of Agriculture, Faisalabad, 38040 Pakistan; 2 Department of Chemistry, College of Science, Princess Nourah Bint Abdulrahman University, 11671 Riyadh, Saudi Arabia; 3 Institute of Horticultural Sciences, University of Agriculture, Faisalabad, 38040 Pakistan

**Keywords:** 28-homobrassinolide, Ethephon, Menthol, *Mentha arvensis* L. LIBS, DSC-TGA, SEM, Natural hazards, Chemical biology, Chemical safety, Environmental chemistry

## Abstract

Nutrient leaching is a major reason for fresh and ground water contamination. Menthol is the major bioactive ingredient of *Mentha arvensis* L. and one of the most traded products of global essential oil market. The indigenous production of menthol crystals in developing countries of the world can prove to be the backbone for local growers and poor farmers. Therefore, present research was designed to check the effects of nano-structured plant growth regulators (PGRs) (28-homobrassinolide and ethephon) with reduced leaching potentials on the essential oil and menthol (%) of *Mentha arvensis* L. The prepared nano-formulations were characterized by Fourier transform infrared (FTIR) spectroscopy, Laser induced breakdown spectroscopy (LIBS), Differential scanning colorimetry-thermal gravimetric analysis (DSC-TGA), Scanning electron microscopy (SEM), Atomic absorption spectrometry (AAS) and Zeta potential and Zeta size analysis. The menthol (%) was determined by modified spectrophotometric and gas chromatographic (GC) method. The highest essential oil (%) was obtained by the application of 28-homobrassinolide-Zn-NPs-L-II (0.92 ± 0.09%) and ethephon-Ca-NPs-L-III (0.91 ± 0.05%) as compared to the control (0.65 ± 0.03%) and blank (0.62 ± 0.09%). The highest menthol (%) was obtained by applying 28-homobrassinolide-Ca-NPs-L-I (80.06 ± 0.07%), 28-homobrassinolide-Ca-NPs-L-II (80.48 ± 0.09%) and 28-homobrassinolide-Ca-NPs-L-III (80.84 ± 0.11%) and ethephon-Ca-NPs-L-III (81.53 ± 0.17%) and ethephon-Zn-NPs-L-II (81.93 ± 0.26%) as compared to control (67.19 ± 0.14%) and blank (63.93 ± 0.17%).

## Introduction

The *Mentha arvensis* L. (menthol mint, wild mild, corn mint, Japanese mint and field mint) is an important aromatic medicinal plant having number of applications in pharmaceutical industries, cosmetic products and food and flavouring agents^1^. The essential oil of *Mentha arvensis* L. contain many bioactive components such as oxygenated monoterpenes, monoterpene hydrocarbons, oxygenated sesquiterpenes, sesquiterpene hydrocarbons, ester, ketone, alcohol and all terpene related compounds^2^. Menthol is the major secondary metabolite of *Mentha arvensis* L.^1^, having analgesic and pain relieving effects, with transient receptor potential melastatin-8 (TRPM8)^3^. According to an estimate, the global essential oil market is valued approximately 10.3 billion USD in 2021 and expected to reach upto 16.0 billion USD by the end of 2026, with the CAGR of 9.3%^4^. As per the data of "Observatory of Economic Complexity", menthol (C_10_H_20_O) is the world’s 2015th most traded product with the total imports and exports of upto millions of USD. As per the recent report of 2020, currently the global menthol market is around 930 Million USD and expected to reach upto 1298.4 Million USD till the end of 2027, with the CAGR of 4.8% during the forecasted period of 2021 to 2027^5^.

The percentage of menthol in *Mentha arvensis* L. varies by varying the harvesting time, geographical location, availability of water, fertility of soil, climate change, biotic and abiotic stresses and soil texture^6^. Therefore, exogenous application of plant growth regulators (PGRs) (including plant growth promoters and plant growth inhibitors) helps to ensure the additional salt stress, for triggering the biosynthesis of secondary metabolites in plants. The nano-structured plant growth regulators have proved to be the most proficient elicitors for stimulating the production of secondary metabolites, with the minimum agricultural input^7^. These natural or synthetic biostimulants not only control the total anti-oxidant compounds of herbaceous plants but also elucidate the physiological responses and biochemical pathways necessary for proper growth and development. Some major types of plant growth regulators are: (i) auxins (indole-3-acetic acid (IAA), indole-3-acetonitrile (IAN), indole-3-acetaldehyde (IAc), ethylindole acetate and indole-3-pyruvic acid (IPyA)) (ii) cytokinins (ribosylzeatin, zeatin, iso-pentinyladenine and dihydrozeatin) (iii) gibberellins (GA_4_, GA_3_ and GA_7_) (iv) ethylene releasing compounds (ethephon and ethrel) (v) brassinosteroids (dolicholide, 28-homodolicholide, casta-sterone, dolichosterone, 28-homodolichosterone and typhasterol) (vi) jasmonates (jasmonic acid) and (vii) strigolactones (strigol, GR_24_ and orobanchol).

Brassinosteroids are steroidal hormones naturally occurring in plants. They induce stress tolerance in aromatic herbs by regulating the production of reactive oxygen species (ROS) in living cells. The exogenous application of 28-homobrassinolide improves catalytic activities of monodehydroascorbate reductase, superoxide dismutase, dehydroascorbate reductase, ascorbate peroxidase, glutathione reductase and catalases. The 28-homobrassinolide also improves the redox potential of naturally occuring anti-oxidant compounds of plants to tolerate abiotic stresses^8^. The ethephon belong to the ethylene releasing compounds that potentially interferes with the growth processes and hormonal systems of plants. Ethephon is a known plant growth retardant (PGR), having growth inhibiting effects. It improves the opening and closing of stomata, induces the sterility of pollens, influences the biosynthesis of secondary metabolites, enhances the herbicidal efficacy, eradicates the pests, and depresses the formation of vesicular arbuscular mycorrhizae. The mode of action of ethephon in plants is quite similar to the mode of action of auxins that involve two step processes including (i) biosynthesis of abscisic acid and (ii) production of ethylene in plants^9–11^.

In view of all the above mentioned discussion, it seems to be essential to trigger the plant growth and its aromatic constituents by external input of PGRs impregnated with essential macro and micro-nutrients for commercial scale utilization of valuable market products. However, there are number of problems which make the entire process quite difficult and unapproachable. Excessive input of agrochemicals and basic plant hormones not only increase the cost of cropping system but also cause the wastage of soil nutrients via leaching. Leaching of chemicals from agricultural land area, lead to the contamination of natural water resources which in turn damage the life quality of aquatic animals and human beings of close vicinity. Water pollution not only affects the living organisms but also the soil quality and entire cropping system. In short, one environmental sphere affects the all other including living and non-living components of extreme importance^12,13^.

Nanotechnology holds great potential in overcoming such type of problems by taking a step forward towards sustainable developments^14^. Use of nano-scale fertillizers and plant growth regulators (PGRs) ensure the targeted delivery, controlled release and minimum input of agrochemicals without wastage^15^. Nano-structured PGRs have more tendencies for better assimilation and high absorption in the roots of plants without degrading through light and high temperature^16–18^. However, extensive review of literature made a conclusion that no such type of nanoformulations have been prepared and reported so far. Previously, most of the PGRs have been used in pure form without considering their harmful environmental consequences. The photo stabilities, thermal stabilities, cytotoxic profiles and leaching potentials have not been considered seriously. The concept of impregnation of PGRs with basic plant nutrients (macro-nutrients, micronutrients and chelating agents) have not been applied anywhere. The minimum input of nanostructured PGRs for maximum productivity of plants, with better assimilation and minimum harmful environmental consequences is the major capital of present study.

Therefore, present research was designed to check the effects of nano-structured PGRs (28-homobrassinolide and ethephon) on the essential oil contents and menthol percentage of *Mentha arvensis* L. The photo stability, thermal stability, leaching potential and cytotoxic effects of prepared nano-formulations was also checked for commercial scale utilization of NPs. All the prepared nano-formulations were characterized by advanced spectroscopic and chromatographic techniques including Fourier transform infrared (FTIR) spectroscopy, Laser induced breakdown spectroscopy (LIBS), Differential scanning colorimetry-thermal gravimetric analysis (DSC-TGA), Scanning electron microscopy (SEM), Atomic absorption spectrometry (AAS), Zeta potential and Zeta size analysis and gas chromatographic (GC) method. These types of nano-formulations can help to alter the traditional cropping system and entire scenario on the global agricultural canvas.

## Materials and methods

### Chemicals and reagents

All the chemicals, reagents, plant growth regulators and gas chromatographic standards used in this research work were of analytical grade. The plant growth regulators including 28-homobrassinolide (C_29_H_50_O_6_) (CAS No. 80483-89-2) and ethephon (C_2_H_6_ClO_3_P) (CAS No. 16672-87-0) were purchased from XI’AN NORSON BIOTECH CO., LTD, China.

The chemicals including hydrochloric acid (HCl) (CAS No. 7647-01-0), sulphuric acid (H_2_SO_4_) (CAS No. 7664-93-9), sodium hydroxide (NaOH) (CAS No. 1310-73-2), disodium edetate dihydrate (Na_2_EDTA⋅2H_2_O) (CAS No. 6381-92-6), sodium metasilicate nonahydrate (Na_2_O_3_Si⋅9H_2_O) (CAS No. 13517-24-3), calcium chloride (CaCl_2_) (CAS No. 10043-52-4), calcium nitrate tetrahydrate (Ca(NO_3_)_2_⋅4H_2_O) (CAS No. 13477-34-4), sodium carbonate (Na_2_CO_3_) (CAS No. 497-19-8), zinc nitrate hexahydrate (Zn(NO_3_)_2_⋅6H_2_O) (CAS No. 10196-18-6), anhydrous sodium sulphate (Na_2_SO_4_) (CAS No. 7757-82-6), mannitol (C_6_H_14_O_6_) (CAS No. 69-65-8), ethylenediaminetetraacetic acid (C_10_H_16_N_2_O_8_) (CAS No. 60-00-4), magnesium chloride (MgCl_2_) (CAS No. 7786-30-3), dipotassium hydrogen phosphate (K_2_HPO_4_) (CAS No. 7758-11-4), sodium nitrate (NaNO_3_) (CAS No. 7631-99-4), dimethyl sulfoxide ((CH_3_)_2_SO) (CAS No. 67-68-5), potassium dihydrogen phosphate (KH_2_PO_4_) (CAS No. 7778-77-0), potassium nitrate (KNO_3_) (CAS No. 7757-79-1), magnesium sulfate (MgSO_4_) (CAS No. 7487-88-9), boric acid (H_3_BO_3_) (CAS No. 10043-35-3), copper sulfate pentahydrate (CuSO_4_⋅5H_2_O) (CAS No. 7758-99-8), zinc sulfate heptahydrate (ZnSO_4_⋅7H_2_O) (CAS No. 7446-20-0), manganese chloride tetrahydrate (MnCl_2_⋅4H_2_O) (CAS No. 13446-34-9), ammonium molybdate dihydrate ((NH_4_)_6_MoO_24_⋅2H_2_O) (CAS No. 12054-85-2), ferrous sulfate heptahydrate (FeSO_4_⋅7H_2_O) (CAS No. 7782-63-0), iron sodium ethylenediaminetetraacetic acid (C_10_H_12_N_2_NaFeO_8_) (CAS No. 15708-41-5), phenol (C_6_H_5_OH) (CAS No. 108-95-2), chloroform (CHCl_3_) (CAS No. 67-66-3), ethanol (CH_3_CH_2_OH) (CAS No. 64-17-5), gallic acid ((HO)_3_C_6_H_2_COOH) (CAS No. 149-91-7), aluminum trichloride (AlCl_3_) (CAS No. 7446-70-0), methanol (CH_3_OH) (CAS No. 67-56-1), potassium ferricyanide (K_3_[Fe(CN)_6_]) (CAS No. 13746-66-2), trichloro acetic acid (Cl_3_CCOOH) (CAS No. 76-03-9) and ferric chloride (FeCl_3_) (CAS No. 7705-08-0) were purchased from Sigma Aldrich.

The reagents including Methylthiazolyldiphenyl-tetrazolium bromide (MTT) reagent (C_18_H_16_BrN_5_S) (CAS No. 298-93-1), Folin-Ciocalteu reagent (CAS No. MFCD00132625), (−)-Epigallocatechin gallate (EGCG) (CAS No. 989-51-5), 1,1-diphenyl-2-picrylhydrazyl (DPPH) (C_18_H_12_N_5_O_6_) (CAS No. 1898-66-4), Phosphate buffer (pH 6.6 at 25 °C) (CAS No. P8165) were purchased from Merck.

The gas chromatographic standards including l-menthol (CAS No. 2216-51-5), menthone (CAS No. 10458-14-7), 1,8-cineol (CAS No. 470-82-6), eugenol (CAS No. 97-53-0), camphor (CAS No. 76-22-2), myrcene (CAS No. 123-35-3), α-pinene (CAS No. 7785-26-4), γ-terpinene (CAS No. 99-85-4), linalool (CAS No. 78-70-6), borneol (CAS No. 464-45-9) and d-menthol (CAS No. 15356-60-2) were purchased from Sigma Aldrich and Merck.

### Preparation of nano-structured PGRs

#### Ethylene diammine tetraacetic acid based nano-particles

The 28-homobrassinolide-EDTA-NPs and ethephon-EDTA-NPs were prepared by separately dissolving 90 g of 28-homobrassinolide (pH = 3–4) and ethephon (pH = 2) in 80 mL and 30 mL of distilled water (reaction temperature: 35 °C, reaction time: 1 h and stirring intensity: 700 rpm). After the 1 h of continuous stirring, 5.364 g of Na_2_EDTA⋅2H_2_O (dissolved in 10 mL of distilled water), was added in the reaction mixtures (reaction temperature: 35 °C, reaction time: 1 h and stirring intensity: 700 rpm). The 28-homobrassinolide formed smooth, creamy and thick off-white paste which changed into thin, off-white and easily stirrable precipitates (pH = 4–5) while ethephon showed no change in physical state except off-white colour changed into pure white grains (pH = 3). After the completion of stirring period, concentrated HCl was added (pH = 2) in the mixtures (reaction temperature: 35 °C, reaction time: 4 h and stirring intensity: 1000 rpm) and let them to stir again. After the 4 h of stirring, the prepared NPs were allowed to settle, upper liquid layers were decanted, and NPs were dried (at 45 °C for about 6 to 8 h) for further use.

#### Silica based nano-particles

The 28-homobrassinolide-Si-NPs and ethephon-Si-NPs were prepared by dissolving 90 g 28-homobrassinolide (pH = 4) and ethephon (pH = 2) separately in 130 mL and 140 mL of distilled water (reaction temperature: 35 °C, reaction time: 1 h and stirring intensity: 700 rpm). After the completion of 1 h, 3.5 g Na_2_O_3_Si⋅9H_2_O was added in the reaction mixtures (pH = 9) and allowed to stir again (reaction temperature: 35 °C, reaction time: 1 h and stirring intensity: 700 rpm). This stirring period lead to the formation of off-white precipitates of 28-homobrassinolide and converted smooth skin paste of ethephon into brownish gelatinous liquid, having shiny appearance. After the 1 h of continuous stirring, highly concentrated H_2_SO_4_ was gradually added in a dropwise manner till the pH = 5.5. In case of 28-homobrassinolide, only few drops changed the colour of granular solution into light green, while no change was observed in the physical state of ethephon. At this pH range, the concentrated solution of CaCl_2_ was added in the mixtures and let them to stir again (reaction temperature: 35 °C, reaction time: 12 h and stirring intensity: 700 rpm). As a result of this stirring, clear solution of 28-homobrassinolide turned into white foamy mixture while clear brown solution of ethephon changed into densely clouded brownish liquid. After 12 h of stirring, dark brownish liquid was converted into light brown solution. These mixtures were centrifuged at 4000 rpm, upper liquid layers were separated and thick precipitates were dried (at 45 °C for about 6 to 8 h) and finely ground.

#### Calcium based nano-particles

The 28-homobrassinolide-Ca-NPs and ethephon-Ca-NPs were prepared by dissolving the 90 g of 28-homobrassinolide (pH = 3–4) and ethephon (pH = 2) in 80 mL and 30 mL of distilled water separately (reaction temperature: 35 °C, reaction time: 1 h and stirring intensity: 700 rpm). After the 1 h of continuous stirring, 850.32 g Ca(NO_3_)_2_⋅4H_2_O (pH = 5) was added in the reaction mixtures (reaction temperature: 35 °C, reaction time: 1 h and stirring intensity: 700 rpm), resulting in the conversion of pale yellow granular solution of 28-homobrassinolide into smooth off-white paste (pH = 2). However, no change was observed in the physical state of ethephon (pH = 2). After this stirring period, 850.32 g of Na_2_CO_3_ (pH = 10) was added in the above mentioned solutions (reaction temperature: 35 °C, reaction time: 1 h and stirring intensity: 700 rpm), resulting in the formation of yogurt like creamy mixture of 28-homobrassinolide (pH = 6) from smooth off-white paste. However, mixture of ethephon reduced the reaction temperature and showed a unique property of instant bubbling after the addition of Na_2_CO_3_, due to the release of carbon dioxide and ethylene gas. After the completion of 1 h, fully concentrated solution of NaOH was added in a dropwise manner, till the pH = 10 and allowed the mixtures to stir again (reaction temperature: 35 °C, reaction time: 18 h and stirring intensity: 700 rpm). In case of ethephon-Ca-NPs, the apparent colour of ethephon changed from champion brown to golden brown and then dark brown to dark black. After 18 h of continuous stirring, both the mixtures were centrifuged at 4000 rpm, decanted and dried (at 45 °C for about 6 to 8 h).

#### Zinc based nanoparticles

The 28-homobrassinolide-Zn-NPs and ethephon-Zn-NPs were prepared by dissolving 90 g 28-homobrassinolide (pH = 2) and ethephon (pH = 2) separately in 70 mL and 30 mL distilled water (reaction temperature: 35 °C, reaction time: 1 h and stirring intensity: 700 rpm). After this period of time, 0.104 g Zn(NO_3_)_2_⋅6H_2_O (pH = 6) was added in the reaction mixtures and let them to stir (reaction temperature: 35 °C, reaction time: 1 h and stirring intensity: 700 rpm). This stirring period led the formation of thick off-white granular solution of 28-homobrassinolide having pH = 3 and converted skin off-white paste of ethephon into easily stirrable quite thin solution (pH = 2). After the 1 h of stirring period, 0.075 g Na_2_SO_4_ (pH = 8) was added in the solutions and let them to stir again (reaction temperature: 35 °C, reaction time: 1 h and stirring intensity: 700 rpm). The 28-homobrassinolide showed no change in physical state while ethephon showed conversion of smoothly stirring off-white solution into turbid mixture. After the completion of stirring, concentrated NaOH was added in the mixture (reaction temperature: 35 °C, reaction time: 15 h and stirring intensity: 700 rpm), resulting in the formation of light green solution (pH = 8) of 28-homobrassinolide and crystal clear pure golden liquid (pH = 9) of ethephon. After the 15 h, both these reaction mixtures were allowed to settle, upper liquid layers were decanted, and skin off-white and dark brown precipitates were dried (at 45 °C for about 6 to 8 h) for further use.

### Preparation of Hoagland solution

The Hoagland solution was prepared by mixing the macro-nutrients, micro-nutrients and complexing agents as per the calculations mentioned in literature^19^.

### Preparation of field and application of doses

The freshly harvested young stems of *Mentha arvensis* L. were collected from Rosa Project of Institute of Horticultural Sciences, University of Agriculture, Faisalabad, Pakistan. These healthy stems were cut into 5 to 7.5 cm pieces and placed into 8 to 10 cm deep furrow at the approximate distance of 45 cm^20^. The young plants were watered at alternate days for 21 days after plantation, prior to the application of doses^1^. After the growth of young plants, both nano-structured PGRs and fixed concentration nutrient media, were applied to the plants at three different concentrations (low: 25 ppm (Level-I), medium: 100 ppm (Level-II) and high: 250 ppm (Level-III)) for consecutive 20 weeks through root application^21^.

### Harvesting and extraction of essential oil

After the completion of dose application and growth of *Mentha arvensis* L., the healthy and matured plants were harvested and shade dried, prior to the extraction of essential oil^22^. The essential oil was extracted through Clevenger type hydrodistillation apparatus^23^. The extracted essential oil was collected in separate containers, dried through anhydrous sodium sulphate, filtered with micro-filters and refrigerated for further analysis. Essential oil (%) was calculated as per the following formula (Eq. 1)^23^:

1 $$\mathrm{Yield \; of \;essential \;oil \,}\left(\mathrm{\%}\right)=\frac{\mathrm{Net \; weight\; of \;essential \;oil \,}({\text{g}})}{\mathrm{Total \;weight\; of \;dried \;biomass \,}({\text{g}})}\times 100$$

### Determination of chemical constituents of essential oil

#### Spectrophotometric determination

All the essential oil samples were subjected to colorimetric assay for the determination of menthol (%) in *Mentha arvensis* L. as per the specifications mentioned in previous studies^24,25^. The standard equation obtained from standard curve of variable concentrations of GC-grade menthol was $y=0.0185x+0.0922$ with ${R}^{2}=0.989$.

#### Gas chromatographic analysis

After the spectrophotometric determination of menthol (%), some selected essential oil samples were subjected to gas chromatographic analysis using BK-GC_7820_ Gas Chromatograph, Biobase, China as per the experimental protocol mentioned in literature^26^.

### Biological activities

#### Total phenolic contents (TPC)

The TPC of aqueous extracts of *Mentha arvensis* L. were determined by experimental protocols as mentioned in previous study^27^. The standard equation obtained by standard curve of gallic acid for the assessment of TPC was ${\text{y}}=0.0137{\text{x}}+0.2987$ with ${{\text{R}}}^{2}=0.9897$.

#### Total flavonoid contents (TFC)

The TFC of aqueous extracts of *Mentha arvensis* L. were determined by experimental protocols as mentioned in previous research^27^. The standard equation obtained by the standard curve of catechin for the assessment of TFC was ${\text{y}}=0.0044{\text{x}}-0.0566$ with ${{\text{R}}}^{2}=0.989$.

#### DPPH free radical scavenging activity

The DPPH free radical scavenging potentials of aqueous extracts of *Mentha arvensis* L. were determined by experimental protocols as mentioned in previous article^27^. The DPPH free radical scavenging potentials (%) were calculated as per the following formula (Eq. 2)^28^:

2 $$\mathrm{DPPH \;scavenging \;activity }\left(\mathrm{\%}\right)=100-\left[\frac{\mathrm{Sample\;absorbance}}{\mathrm{Control \;absorbance}}\right]\times 100$$

#### Reducing power ability (RPA)

The RPA of aqueous extracts of *Mentha arvensis* L. were determined by experimental protocols as mentioned in literature^27^. The standard equation obtained by the standard curve of gallic acid for the assessment of RPA was ${\text{y}}=0.0114{\text{x}}-0.0455$ with ${{\text{R}}}^{2}=0.989$.

### Assessment of physiochemical characteristics

#### Photodegradation

The photodegradation profiles of 28-homobrassinolide, ethephon and their corresponding NPs were determined by exposing 250 ppm solutions to the natural sunlight, for about 8 to 10 h daily at 35 °C upto 28 days. The UV–Vis spectra were recorded from 340 to 800 nm at (i) day 0 (ii) day 1 (iii) day 3 (iv) day 5 (v) day 7 (vi) day 14 and (vii) day 28, using 721(D) spectrophotometer, China^29–31^.

#### Thermal degradation

The thermal degradation profiles of 28-homobrassinolide, ethephon and corresponding NPs were determined by heating 250 ppm solutions at variable temperatures, for 8 to 10 h daily. The UV–Vis spectra were recorded from 340 to 800 nm at (i) 30 °C (ii) 35 °C (iii) 40 °C (iv) 45 °C (v) 50 °C (vi) 55 °C and (vii) 60 °C, using 721(D) spectrophotometer, China^32,33^.

#### Leaching potential

The leaching potentials of 28-homobrassinolide, ethephon and their corresponding NPs were determined by adding the 50 mL of 250 ppm solution in a glass column, having the internal diameter of 20 mm, filled with sand (particle size: 0.05 mm to 2.00 mm), silt (particle size: 0.002 mm to 0.05 mm) and clay (particle size: ˂0.002 mm). The standard equations obtained from standard curves of 28-homobrassinolide, ethephon and their corresponding NPs at variable concentrations were having ${{\text{R}}}^{2}>0.99$. The obtained leachate solutions were also analyzed by 721(D) spectrophotometer, China at the λ_max_ of NPs^34^.

#### MTT cytotoxic assay

##### Isolation of living cells

The MTT cytotoxic assay was performed by isolating the living cell suspensions from healthy leaves of *Mentha arvensis* L. as per experimental protocols mentioned in previous research article^35^.

##### Method of preparation of negative control (NC)

The negative control was prepared by obtaining the standard equation from standard curve $({\text{y}}=0.0441{\text{x}}+0.481\mathrm{ with }{{\text{R}}}^{2}=0.9914)$ using variable concentration of living cell suspensions as per the experimental protocols mentioned in literature^36^.

##### Method of preparation of positive control (PC)

The positive control was prepared by adding variable concentrations of nano-structured PGRs (25, 50, 75, 100, 125, 150, 175, 200, 225 and 250 ppm) in 2 μL of living cell suspensions, followed by the shaking at 200 rpm. All the next steps were according to the experimental protocols as mentioned in previous studies^36,37^. The viable cell concentrations (%) were calculated according to the following equation (Eq. 3)^36,37^:

3 $$\mathrm{Cell\; viability }\left(\mathrm{\%}\right)=\frac{{{\text{PC}}}_{\left({\text{Absorbance}}\right)}-{{\text{NC}}}_{\left({\text{Absorbance}}\right)}}{{{\text{PC}}}_{\left({\text{Absorbance}}\right)}}\times 100$$

### Characterization techniques

#### Fourier transform infrared spectroscopy (FTIR)

The functional groups of 28-homobrassinolide, ethephon and their corresponding NPs were determined by using Agilent Cary 630 FTIR spectrometer, USA as per the experimental protocols mentioned in literature^38^. The FTIR is used to identify the chemical bonds of organic and inorganic molecules by producing infrared absorption spectrum. The spectrum obtained by the FTIR analysis helps to produce a sample profile having distinctive molecular fingerprints. These fingerprints can help to scan and screen the samples with many different chemical constituents. FTIR is an effective analytical tool for determination of functional groups and identification of covalently bonded molecules. In the present study, all the solid samples were ground with the IR transparent potassium bromide (KBr) and strongly pressed to form the pellets for further analysis^39^.

#### Laser induced breakdown spectroscopy (LIBS)

The elemental analysis of 28-homobrassinolide, ethephon and their corresponding NPs was performed by using LIBS: model Q-Smart 850 and NOVA Quantal, France as per the instrumental specifications and experimental protocols mentioned in previous research^40^. LIBS is an advanced analytical technique used to identify the elements in organic and inorganic samples. The elemental composition of materials can be done by using high focused laser for surface ablation. As a result of ablation, momentary plasma is generated consisting of excited atoms, ions and electrons. When these excited species returned back to their ground state, the characteristic wavelengths are produced named as "unique fingerprints". These fingerprints are used for both qualitative and quantitative analysis. In the present study, all the solid samples were directly subjected to LIBS analysis by depositing the nanoparticles on the flat surface of sample holder^41^.

#### Differential scanning colorimetry-thermogravimetric analysis (DSC-TGA)

The thermal stability of 28-homobrassinolide and ethephon was determined by using SDT Q_600_ V_20.9_ Build 20, Crawley, UK as per the instrumental specifications mentioned in previous study^42^. Thermogravimetric analysis (TGA) is used to check the thermal stability of organic and inorganic samples as it deals with the study of gradual weight loss in sample with respect to the change in temperature in a very controlled manner. Similarly, differential scanning calorimetry (DSC) can track the changes involved in the flow of heat from sample with the gradual change in temperature in a controlled environment. This technique provide useful information about endothermic and exothermic behaviours, melting temperature, clearing temperature and degradation temperature which helps to determine the extent of heating of sample during the preparation of nanomaterials. In the present study, solid samples were only dried prior to the thermal analysis and directly placed on the sample holder to study the weight loss with respect to the change in temperature^43^.

#### Scanning electron microscopy (SEM)

The surface characteristics and morphological properties of 28-homobrassinolide-Ca-NPs and ethephon-Zn-NPs were determined by Nova NanoSEM 450, FEI, Oregon, USA as per the experimental protocols and instrumental specifications mentioned in literature^44^. SEM is widely used to provide information about size of nanoparticles and morphological characteristics of smaller materials. In SEM analysis, a fast moving beam of electron is focused on the surface of sample which interacts with the atoms of sample to provide three-dimensional topographical information about the surface^45^. Sample preparation is a very crucial step during the SEM analysis for accurate results as sample should be completely dry and conductive. The moistured sample creates problems during the analysis as residual water reduces the quality of images. In order to determine the sample characteristics more accurately, the cleaned analytical samples were thoroughly dehydrated before mounting on the surface of stub. The non-conductive dried samples were also coated with the layer of chromium based conductive material by optimizing the thickness of coating materials upto 10 nm^46^.

#### Atomic absorption spectroscopy (AAS)

The trace metal analysis of 28-homobrassinilide-Zn-NPs and ethephon-Zn-NPs were performed by using Z-8200 polarized Zeeman atomic absorption spectrophotometer (AAS) Hitachi, Japan with instrumental specifications as mentioned in previous research article^47^. AAS is a well-known conventional analytical technique used for the quantitative determination of elements in a wide range of analytical samples with good reliability and simplicity. It works by absorbing the specific wavelength of light for excitation of electrons followed by the de-excitation and production of specific pattern of wavelengths. In the present study, calibrated standards were prepared from the commercially available stock solution (Applichem^®^) in the form of an aqueous solution (1000 ppm). Highly purified de-ionized water was used for the preparation of working standards. All the glass apparatus used throughout the process of analytical work were immersed in 8N HNO_3_ overnight and washed with several changes of de-ionized water prior to use. The samples were directly analyzed and compared^47^.

#### Zeta potential and zeta size analysis

The zeta potential and zeta size analysis of nanostructured PGRs (28-homobrassinolide-EDTA-NPs, 28-homobrassinolide-Si-NPs, 28-homobrassinolide-Ca-NPs, 28-homobrassinolide-Zn-NPs, ethephon-EDTA-NPs, ethephon-Si-NPs, ethephon-Ca-NPs and ethephon-Zn-NPs) were analyzed by using Zetasizer, Malvern Zetasizer Nano ZSP, UK. The zeta potential and zeta size analysis helps to determine the particle size distribution, mobility of nanoparticles, surface characteristics, concentration of sample and molecular weights of polymeric structures. Zetasizer works on the principle of "Dynamic Light Scattering" (DLS) having the particle size range of 0.3 nm to 10 microns (diameter). In the present study, all the solid samples were dispersed in the water having refractive index: 1.330, viscosity: 0.8872 and dielectric constant: 78.5. For the zeta potential analysis, working temperature of Zetasizer was 25.1 °C, Zeta runs: 28, count rate (kcps): 448.6, measurement position (mm): 2.00, cell dispersion: clear disposable zeta cell and attenuator: 6. For the zeta size analysis, working temperature of Zetasizer was 25.1 °C, duration used: 60 s, count rate (kcps): 330.1, measurement position (mm): 5.50, cell dispersion: clear disposable zeta cell and attenuator: 9^48^.

### Statistical analysis

The highest menthol (%) of *Mentha arvensis* L., grown by applying three different concentrations (low: 25 ppm (Level-I), medium: 100 ppm (Level-II) and high: 250 ppm (Level-III)) of nano-structured PGRs were statistically compared by using ANOVA along with post-hoc Tukey HSD test^49^.

## Results and discussion

### Physical attributes and percentage yield of essential oil

In the present study, *Mentha arvensis* L. was grown by the application of nano-structured PGRs (EDTA-NPs, Si-NPs, Ca-NPs and Zn-NPs) at three different dose concentrations [Level-I: 25 ppm, Level-II: 100 ppm and Level-III: 250 ppm], to check their effects on the yield and menthol (%) of essential oil. The results of present study showed that essential oil yield of *Mentha arvensis* L., grown by applying the above mentioned nano-formulations ranged from 0.92 ± 0.09 to 0.65 ± 0.09%. The highest essential oil yield was obtained by the *Mentha arvensis* L., grown by the application of 28-homobrassnolide-Zn-NPs-L-II as brassinosteroids and all structural analogues are known to have significant influence on the physiological attributes and biochemical processes of aromatic medicinal plants. These steroidal hormones have been reported to regulate the photosynthetic processes^50^ and catalytic potentials of several enzymes^51^.

According to previous study^52^, foliar spray of 10^–7^ M 28-homobrassinolide remarkably increased the percentage of menthyl acetate (225.0–187.5%), menthol (135.9–134.1%) and menthone (180.0–161.1%) in the essential oil of *Mentha arvensis* L. It has also been reported by researchers^53^ that *Mentha arvensis* L. showed higher biomass yield and improved essential oil (%) (including menthol, l-menthone, isomenthone and menthyl acetate) at 10^−7^ M concentration of 28-homobrassinolide as compared to lower (10^–6^ M) and higher (10^–8^ M) concentrations. In addition to the plant growth regulator (PGR), Zn-NPs itself act as an important growth promoter of the aromatic medicinal herbs, by enhancing the nutrient absorption capacity of number of essential macro-nutrients. In some previous studies^54,55^, researchers reported the similar effects on essential oil contents of *Ocimum sanctum* and *Mentha piperita* by the exogenous application of zinc. In another study^56^, scientists mentioned the 4.73% increase in menthol (%) in essential oil of menthol mint at 5.0 mg Zn kg^−1^ application of doses.

### Determination of chemical constituents of essential oil

#### Spectrophotometric determination

As per the results obtained by present research, menthol percentage of essential oil of *Mentha arvensis* L., grown by applying all the above mentioned nano-particles were in the range of 81.93 ± 0.26 to 54.01 ± 0.04%. The highest menthol contents were obtained by the essential oil of *Mentha arvensis* L., grown by application of ethephon-Zn-NPs-L-II as ethylene releasing auxin based compounds show positive synergism in improving the production of menthol^9^. According to previous study^57^, the essential oil of pepper-mint showed significant increase in percentage of isomenthone and neoisomenthol by the foliar application of 250 ppm ethephon. Similarly^58^, previous researchers mentioned the higher biosynthesis of terpenoids in *Cannabis sativa* L., upon exogenous application of 100 µM ethephon. As per the data reported by recent researchers^9^, ethephon provides abiotic stress to the aromatic plants and triggers the biosynthesis of abscisic acid, resulting in the higher production of essential oil. Additionally, Zn-NPs are also known to trigger the production and release of essential oil with higher menthol percentage as zinc activates the anti-oxidant enzymes and stress proteins under abiotic stresses such as excessive salt and severe water shortage^59^.

#### Gas chromatographic analysis

The major and minor chemical constituents of essential oil of *Mentha arvensis* L., grown by applying 28-homobrassinolide-Ca-NPs-L-III and ethephon-Zn-NPs-L-II are shown (Table 1). According to the gas chromatographic analysis, l-menthol is a major chemical constituent of essential oil of *Mentha arvensis* L., with menthone, 1,8-cineol, eugenol, camphor, myrcene, α-pinene, γ-terpinene, linalool, borneol and d-menthol being the minor one. The menthol (%) of *Mentha arvensis* L., grown by the application of 28-homobrassinolide-Ca-NPs-L-III (82.18 ± 0.05%) and ethephon-Zn-NPs-L-II (84.64 ± 0.02%) is much higher as compared to the control (67.23 ± 0.13%) and blank (63.98 ± 0.16%). In a previous study^60^, researchers also reported the higher menthol (38.27%) and menthone (29.87%) contents in essential oil of *Mentha piperita* L., by the exogenous application of 24-epibrassinolide (24-eBL). In another study^52^, scientists reported the 135.9% increase in the menthol production in corn mint (*Mentha arvensis* L.), by the foliar application of three different PGRs including 28-homobrassinolide.

**Table 1:** Gas chromatographic analysis of essential oil of *Mentha arvensis* L.

Sr. no.	Chemical constituents	Essential oil of *Mentha arvensis* L. grown with the application of PGRs			
		28-Homobrassinolide-Ca-NPs-L-III	Ethephon-Zn-NPs-L-II	Control treatment	Blank
		Percentage composition of chemical constituents			
Major chemical constituents					
1	l-Menthol	82.18 ± 0.05	84.64 ± 0.02	67.23 ± 0.13	63.98 ± 0.16
2	Menthone	0.07 ± 0.07	0.0009 ± 0.09	4.45 ± 0.04	0.94 ± 0.04
3	1,8-Cineol	0.60 ± 0.04	2.69 ± 0.01	10.11 ± 0.05	2.59 ± 0.01
Minor chemical constituents					
1	Eugenol	0.05 ± 0.01	0.003 ± 0.04	1.03 ± 0.03	1.71 ± 0.05
2	Camphor	0.07 ± 0.04	0.0009 ± 0.02	2.69 ± 0.07	1.31 ± 0.08
3	Myrcene	16.40 ± 0.05	5.50 ± 0.04	3.91 ± 0.06	22.99 ± 0.03
4	α-Pinene	0.13 ± 0.08	0.12 ± 0.02	0.06 ± 0.15	0.95 ± 0.02
5	γ-Terpinene	0.18 ± 0.09	1.03 ± 0.09	1.78 ± 0.10	0.89 ± 0.06
6	Linalool	0.05 ± 0.02	0.39 ± 0.05	2.81 ± 0.03	3.94 ± 0.10
7	Borneol	0.29 ± 0.01	5.34 ± 0.01	5.93 ± 0.08	0.56 ± 0.06
8	d-Menthol	0.0004 ± 0.03	0.29 ± 0.08	0.0023 ± 0.07	0.14 ± 0.09
Total percentage		100.00	100.00	100.00	100.00

In some previous researches^57–63^, researchers also reported the highly positive effects of ethephon (Phosfon D) spray on the essential oil production and menthol (%) of different aromatic plants. In addition to PGRs, zinc has also been reported to enhance the biosynthesis of menthol in herbaceous plants^56–65^. In addition to the l-menthol, some exceptions have been observed in other chemical constituents of essential oil of *Mentha arvensis* L., such as (a) 28-homobrassinolide-Ca-NPs-L-III (myrcene: 16.40 ± 0.05%) (b) ethephon-Zn-NPs-L-II (borneol: 5.34 ± 0.01%) (c) control (menthone: 4.45 ± 0.04%, 1,8-cineol: 10.11 ± 0.05% and myrcene: 5.93 ± 0.08%) and (iv) blank (myrcene: 22.99 ± 0.03% and linalool: 3.94 ± 0.10%). According to the literature^2^, these variations in the essential oil constituents might also be due to the climatic conditions, seasonal variations and number of biotic and abiotic stresses, capable of altering the biosynthetic pathways of aromatic medicinal herbs.

### Biological activities

#### Total phenolic contents (TPC)

The results of present study showed that the total phenolic contents of aqueous extracts of *Mentha arvensis* L., grown by applying different types of nano-formulations ranged from 50.41 ± 0.02 to 33.11 ± 0.03 mg/L of GAE. The highest total phenolic contents were obtained by the *Mentha arvensis* L., grown by application of ethephon-Si-NPs-L-I as ethylene releasing compounds have been reported to enhance the production of phenols in plants. According to previous study^66^, exogenous application of ethephon caused 82% and 20% increase in anthocyanins and 24 hydroxycinnamic acid in hairy root cultures of black carrot. It has also been reported by researchers^67^ that foliar application of ethephon remarkably increased the total phenolic contents in tempranillo grapes. It has been reported by researchers^68^ that 1000 ppm solution of ethephon increased the total phenolic contents of *Citrus limon* L. According to the literature^69^, *Beta vulgaris* L. exhibited the higher production of total phenolic contents by applying the ethephon. In addition to the ethephon, some additional impacts might also be attributed to the presence of silica nano-particles as silica possesses enough potential to alter the hormonal system of plants. The group of researchers^70^ hold the view that silica improve the catalytic potential of glucose-6-phosphate dehydrogenase in Mung bean sprouts.

#### Total flavonoid contents (TFC)

In the present study, the aqueous extracts of *Mentha arvensis* L., grown by applying all the above mentioned nano-formulations showed the total flavonoid contents ranging from 136.36 ± 0.01 to 14.12 ± 0.12 mg/L of CE. The highest total flavonoid contents were obtained by the aqueous extract of *Mentha arvensis* L., grown by the application of 28-homobrassonolide-L-II because moderate level of plant growth regulators can trigger the biosynthesis of secondary metabolites, by altering the physiological responses in *Lamiaceae*^71^. According to researchers^72^, 1.0 mg L^−1^ dose concentration of 24-epibrassinolide (24-eBL) produced the highest flavonoid contents in transgenic hairy roots of *Echinacea purpurea* L. Moench. There are also some evidences to suggest that 0.0 to 0.5 μM dose concentration of 24-epibrassinolide (EBL) can trigger the production of secondary metabolites in dragonhead (*Dracocephalum moldavica* L.)^73^. Many scholars hold the view that 28-homobrassinolide and all structural analogues can reduce the biotic and abiotic stresses in medicinal plants by altering the hormonal responses and producing the higher quantities of anti-oxidant compounds^74–77^.

#### DPPH free radical scavenging activity

The obtained results showed that DPPH free radical scavenging potentials (%) of aqueous extracts of *Mentha arvensis* L., grown by applying the above mentioned PGRs ranged from 87.78 ± 0.17 to 20.25 ± 0.06%. Both 28-homobrassonolide-Si-NPs-L-I and 28-homo-brassonolide-Zn-NPs-L-III showed significant improvements in DPPH free radical scavenging potentials because the additional input of brassinosteroids (as steroidal hormones) stimulate the production of osmotic constituents and anti-oxidant compounds in plants. It also regulates the phytohormones and stress responses in aromatic medicinal herbs^78^. It has been reported that exogenous application of homobrassinolide enhanced the DPPH free radical scavenging potentials of methanolic extracts of *Mentha arvensis* L. upto 35.83%^79^. The findings of present research work are also supported by the previous study^80^, dealing with the solvent based extraction (combination of ethylene glycol and choline chloride as deep eutectic solvents) of anti-oxidant compounds. According to literature^50^, higher concentrations of 28-homo-brassinolide can enhance the DPPH free radical scavenging activities of *Mentha arvensis* L., due to the increased catalytic potentials of carbonic anhydrases.

#### Reducing power ability (RPA)

In the present research work, the reducing power abilities of aqueous extracts of *Mentha arvensis* L., grown by applying the above mentioned nano-formulations ranged from 92.37 ± 0.23 to 16.55 ± 0.01 mg/L of GAE. The highest reducing power ability was exhibited by the aqueous extract of *Mentha arvensis* L., grown by application of ethephon-L-I as lower concentration of ethylene releasing compounds trigger the hormonal system of plants, responsible for producing the anti-oxidants and phytochemicals^81^. According to previous study^82^, *Solanum melongena* L. also showed the significant variations in anti-oxidant compounds by the exogenous application of 0.15% 2-CEPA (ethephon). According to recent research, exogenously applied ethylene releasing ethephon (2-chloroethyl phosphonic acid) significantly improved the anti-oxidant enzymatic system and number of photosynthetic processes in rice (*Oryza sativa* L.)^83^. There is another evidence to suggest that ethephon (source of ethylene) induces higher production of secondary metabolites, more specifically anti-oxidant compounds in black carrot hairy root culture^66^.

### Assessment of physiochemical characteristics

#### Photodegradation

The photodegradation profiles of nano-structured PGRs showed that almost all the NPs are highly photo-stable. All these chemical compounds showed no change in the UV–Vis spectra ranging from 340 to 800 nm, indicating the longer shelf-life with maintenance of actual structure. Only the small changes were observed in the UV–Vis spectrum of ethephon-Zn-NPs ranging from 340 to 380 nm. These effects might be attributed to the π → π* and n → π* transitions of phosphorous based functional groups of ethephon. As per the data obtained by "Environmental Protection Agency" (EPA), the ethephon (C_2_H_6_ClO_3_P) is a highly resistant compound of nature, and shows only slight structural variations when come in contact with the living organisms of soil, under aerobic conditions^84^. However, ethephon-Zn-NPs showed only slight variations and these effects can be due to the additional input of nano-structured zinc. In a recently conducted research^85^, researchers mentioned the significant improvement in thermal stabilities, chemical characteristics and photocatalytic properties of zinc at nano-scale.

#### Thermal degradation

**Figure 1:**
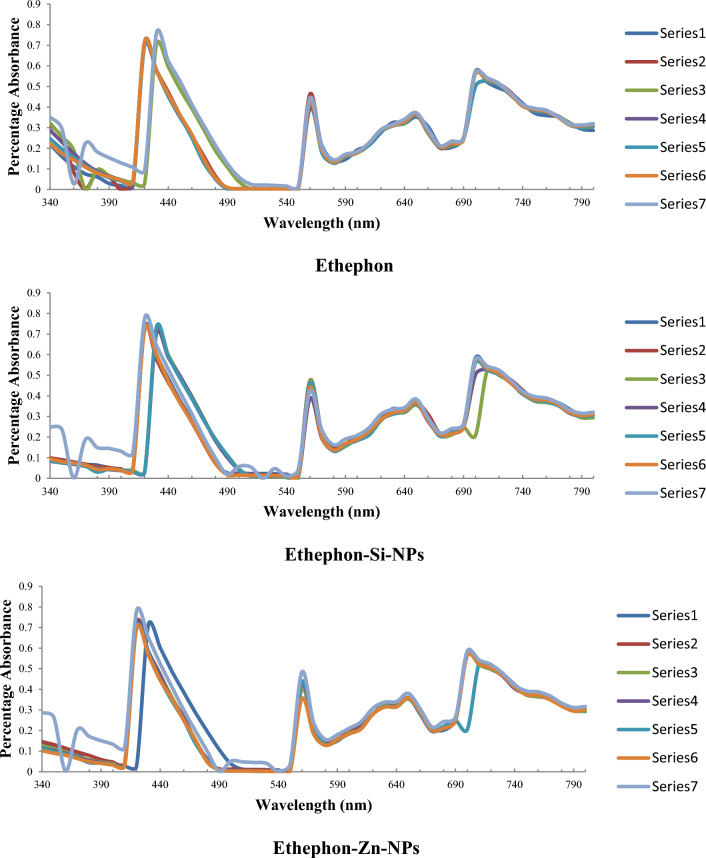
Thermaldegradation profile of ethephon, ethephon-Si-NPs and ethephon-Zn-NPs.

Thermaldegradation profile of ethephon, ethephon-Si-NPs and ethephon-Zn-NPs are shown (Fig. 1). The thermal degradation profiles of nano-structured PGRs showed that all the nano-formulations are highly thermally stable except for the few as mentioned here in detail. The UV–Vis spectrum of ethephon showed minor variations, ranging from 340 to 400 nm, due to the release of ethylene gas. In a previous research article^86^, researchers reported the higher thermal stabilities of ethylene releasing plant growth inhibitors that lead to the formation of di-hydrogen phosphate under alkaline conditions. In case of ethephon-EDTA-NPs, only slight fluctuations were observed in the UV–Vis spectrum ranging from 340 to 390 nm. However, these transitions are very less as compared to the pure ethephon, due to the higher thermal stabilities of EDTA-NPs. In a previously published research^87^, researchers reported the higher thermal stability of EDTA due to its degradation temperature ranging from 200 to 260 °C. During the decomposition of EDTA, only the ethylenic bond (C–N) breaks to produce two stable compounds including (i) N-(2-hydroxyethyl)iminodiacetic acid and (ii) iminodiacetic acid.

Similarly, UV–Vis spectrum of ethephon-Si-NPs also showed some variations in the pattern ranging from 340 to 540 nm. These minor changes indicated the higher thermal stability of silica based nano-formulations. In a recently conducted research^88^, researchers reported the higher thermal stability of calcium silicate hydrate (CaO-SiO_2_-Cr(NO_3_)_3_-H_2_O) upto > 550 °C. The calcium silicate hydrate are thermally stable up-till 500 °C, and starts to change the physical properties and chemical composition at the temperature greater than 550 °C. In the case of ethephon-Zn-NPs, slight variations were observed in the UV–Vis spectrum ranging from 340 to 390 nm. However, these variations were less pronounced as compared to the ethephon, due to the presence of Zn-NPs. In a previously conducted research^89^, researchers reported the higher thermal stabilities of zinc sulphate heptahydrate (ZnSO_4_⋅7H_2_O) upto 120 °C. This molecule only shows the slight variations in weight, due to the loss of water molecules, at a temperature ranging from 70 to 120 °C. By further heating the compound till 260 °C, more weight loss was due to the release of sulphate ions from zinc sulphate.

#### Leaching potential

The leaching potentials of 28-homobrassinolide, ethephon, 28-homobrassinolide-EDTA-NPs, ethephon-EDTA-NPs, 28-homobrassinolide-Si-NPs, ethephon-Si-NPs and 28-homo-brassinolide-Ca-NPs were according to the pore size and empty spaces of soil media. However, some exceptions were observed in the ethephon-Ca-NPs, 28-homobrassinolide-Zn-NPs and ethephon-Zn-NPs. In all these above mentioned nano-formulations, silt showed more affinity for nano-structured PGRs as compared to the clay particles. In case of ethephon-Ca-NPs, ethephon showed lower leaching potentials and higher binding affinities with the silt grains, due to the bond formation in between calcium oxide and silicon oxide as reported in previous research^90^. In the case of zinc based nano-formulations, both 28-homobrassinolide-Zn-NPs and ethephon-Zn-NPs showed maximum retention inside the glass column, with the minimum wastage of all applied chemicals. These effects might be attributed to the fact that zinc can form the divalent cations and chelated metal ions when found in aqueous suspension, resulting in the formation of larger complexes^91^.

#### MTT cytotoxic assay

The obtained result showed that the average cell survival rate of *Mentha arvensis* L., treated with 28-homobrassinolide and ethephon based nano-formulations was 74.28 ± 0.02 and 74.80 ± 0.03%, respectively. The order of viable cell concentration (%) was as follows: (a) 28-homobrassinolide-Zn-NPs (75.10 ± 0.37) > 28-homobrassinolide-Si-NPs (75.01 ± 0.27) > 28-homobrassinolide-Ca-NPs (74.70 ± 0.30) > 28-homobrassinolide (74.08 ± 0.59) > 28-homobrassinolide-EDTA-NPs (72.52 ± 0.28%) and (b) ethephon-Si-NPs (78.02 ± 0.35) > ethephon (74.85 ± 1.61) > ethephon-Ca-NPs (74.16 ± 0.47) > ethephon-Zn-NPs (73.64 ± 0.82) > ethephon-EDTA-NPs (73.31 ± 0.54%). In a previous study^92^, researchers recommended the 10^−7^ M homobrassinolide spray on *Mentha arvensis* L., along with the other plant growth regulators such as triacontanol and irradiated sodium alginates. In another study^52^, researchers also reported the 10^−7^ M 28-homobrassinolide to be optimum for the *Mentha arvensis* L.

In a previously published article^53^, scientists used the variable concentrations of 28-homobrassinolide (10^−0^, 10^−8^, 10^−7^ and 10^−6^ M) and reported the highest menthol (%) by applying 10^−7^ M 28-homobrassinolide. In another research work^60^, researchers mentioned the use of 0.5, 1.5 and 2.5 ppm solutions of 24-epibrassinolide (24-eBL) on the *Mentha piperita* L. In the previously conducted research^57^, group of researchers used the 250 ppm solution of ethephon on the *Mentha arvensis* L. (peppermint), and reported the significant reduction in essential oil (%). In another article^93^, researchers used 0.06% ethephon (2-chloroethyl phosphonic acid) on the Japanese mint and claimed the insignificant variations on the essential oil contents. In another research work^58^, researchers used 1 to 100 µM ethephon solution on the *Cannabis sativa* L. and reported the significant variations in essential oil contents, by varying the concentration of PGRs.

The above mentioned results are compared with the previous literature and data is compiled in tabulated form (Table 2).

**Table 2:** Comparative analysis of obtained results with previous literature.

Sr. no.	Name of PGRs/nanoparticles used in study	Percentage essential oil/menthol contents of current study	Percentage essential oil/menthol contents of previous study	References
1	28-Homobrassinolide	The menthol percentage of essential oil of *Mentha arvensis* L. was increased upto 15.79% at 25 ppm concentration of 28-Homobrassinolide as compared to the blank treatment	The combined application of carrageenan, triacontanol and 28-homobrassinolide increased the menthol percentage in *Mentha arvensis* L. by 6.8 and 7.4% at 100 and 120 days after plantation, respectively	^52^
2	28-Homobrassinolide	The percentage of menthol in *Mentha arvensis* L. was increased upto 14.13% at 100 ppm concentration of pure 28-homobrassinolide as compared to the blank treatment	Gamma-rays irradiated sodium alginate, triacontanol and 28-homobrassinolide collectively enhanced the menthol contents in *Mentha arvensis* L. upto 7.5 and 6.2% at 100 and 120 days after plantation	^92^
3	28-Homobrassinolide	The menthol contents in essential oil of *Mentha arvensis* L. were enhanced upto 10.4% upon root application of 250 ppm solution of pure 28-homobrassinolide in comparison with control treatment where plants were applied with Hoagland solution only	Among the four different concentrations of 28-homobrassinolide, 10^–7^ M concentration enhanced the menthol contents of *Mentha arvensis* L. by 6.9 and 7.2% at 100 and 120 days after plantation as compared to the control	^50^
4	Ethephon	The menthol contents in the essential oil of *Mentha arvensis* L. were improved by 3.66% only as compared to the blank	At the concentration of 0.06%, ethephon showed little effect on the menthol contents of *Mentha arvensis* L. as compared to the control	^61^
5	Ethephon	In the present study, the application of 250 ppm solution of pure ethephon also reduced the menthol contents in essential oil of *Mentha arvensis* L. upto 0.7% as compared to blank	Ethephon at the concentration of 250 ppm reduced the menthol contents in essential oil of *Mentha piperita*	^57^
6	28-Homobrassinolide-EDTA-NPs	The combined application of 25 ppm solution of 28-homobrassinolide impregnated with EDTA-NPs showed 12.39% increase in menthol percentage of *Mentha arvensis* L. as compared to the control treatment	Previous research claimed 134.1% increase in menthol (%) of *Mentha arvensis* L. as compared to control treatment, by applying carrageenan, triacontanol and 28-homobrassinolide. The presence of EDTA-NPs slightly reduced the production of menthol, as EDTA has been reported to lower the catalytic efficiencies of oxidases and dehydrogenases	^50,52,57,61,92,120^
7	Ethephon-Si-NPs	The combined application of ethephon and Si-NPs showed 17.01% increase in menthol percentage of *Mentha arvensis* L. at the concentration of 250 ppm through root application of doses	The ethephon provides the abiotic stress to plants, thereby triggering the biosynthesis of abscisic acid (ABA) and release of essential oil. In addition to the PGR, silica improves the enzymatic activities of nitrate reductase that plays a significant role in the conversion of (−)-iso-piperitenone into (−)-menthol and (±)-neomenthol	^9,121,122^
8	28-Homobrassinolide-Ca-NPs	The combination of 28-homobrassinolide and Ca-NPs showed 16.91% increase in concentration of menthol in essential of *Mentha arvensis* L. by applying 250 ppm solution as compared to the blank treatment	The significant increase in the menthol contents of *Mentha arvensis* L. (135.9% and 161.1% at 100 and 120 DAP) have also been reported in a previous research, dealing with the use of foliar applications of 28-homobrassinolide (HBR). In addition, calcium plays a significant role in intercellular responses, essential for maintaining the integrity of cell membrane and proper cytoplasmic streaming	^52^
9	Ethephon-Ca-NPs	The combination of ethephon and Ca-NPs at 250 ppm, showed 17.6% increase in menthol percentage of *Mentha arvensis* L. as compared to the blank treatment	Ethephon stimulates the enzymatic activities by releasing ethylene gas and synergistic effects of ethephon and Ca-NPs are known to trigger the enzymatic potentials of α-amylase, superoxide dismutase and catalase in aromatic plants	^9,122,123^
10	28-Homobrassinolide-Zn-NPs	The application of 28-homobrassinolide impregnated with Zn-NPs showed 8.65% increase in concentration of menthol in essential oil of *Mentha arvensis* L. as compared to the blank	In a previous study, zinc has been reported to play an important role in stimulation of stress proteins and anti-oxidant enzymes under drought stress and excessive salt accumulation that leads to the higher production of secondary metabolites in plants	^59^
11	Ethephon-Zn-NPs	The combined application of ethephon and Zn-NPs showed 18% higher menthol contents in essential oil of *Mentha arvensis* L. as compared to blank plants	The Zn-NPs proved to be the best in stimulating the production of menthol in *Mentha arvensis* L. because zinc has been reported to increase the concentration of menthone in *Mentha pulegium* L	^124^

### Characterization techniques

#### Fourier transform infrared spectroscopy (FTIR)

**Figure 2:**
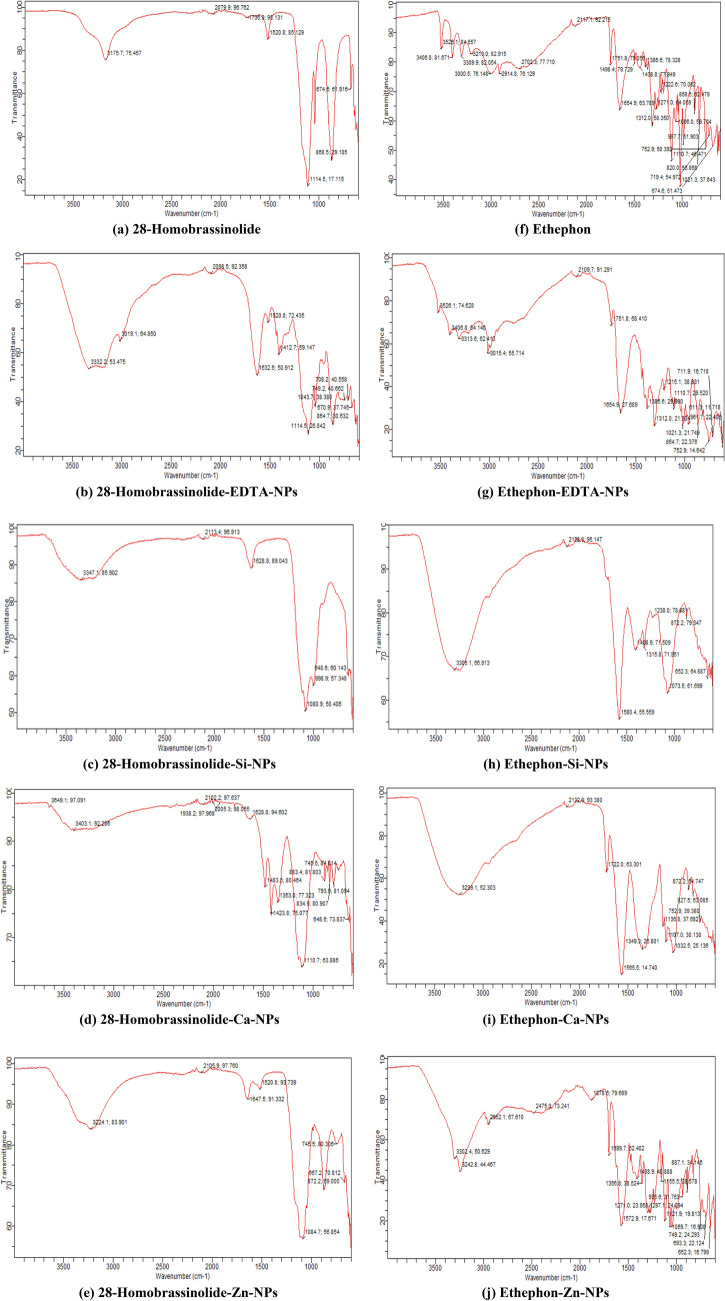
Fourier transform infrared (FTIR) spectroscopic analysis. EDTA-NPs: ethylene diammine tetraacetic acid based nanoparticles, Si-NPs: silica based nanoparticles, Ca-NPs: Calcium based nanoparticles and Zn-NPs: zinc based nanoparticles.

The FTIR spectra of 28-homobrassinolide, ethephon and their corresponding NPs are shown (Fig. 2). In the FTIR spectrum of 28-homobrassinolide, less dominant and weaker peaks at 1736.9 and 2079.9 cm^−1^ were due to the C=O stretching vibrations and C–H bending vibrations of aromatic ringed structure. The tapered ended, dominant, sharp and strong peak at 1114.5 cm^−1^ represented the C–O stretching vibrations of 28-homobrassinolide. In the fingerprint region of FTIR spectrum, the sharp and broad peaks at 674.6 and 868.5 cm^−1^ represented the C=C bending vibrations, produced via liberation of methyl and hydroxyl groups^94^. In the FTIR spectra of 28-homobrassinolide based nano-formulations, only characteristic peaks are described in detail and rest of the peaks are according to the FTIR spectrum of 28-homobrassinolide. In the FTIR spectrum of 28-homobrassinolide-EDTA-NPs, the strong, broad and deep shouldered peak at 3019.1 cm^−1^ indicated the strong stretching vibrations of N–H bonds of amines and the broad and strong peak at 1632.6 cm^−1^ represented the intense C=O stretching vibrations. The two broad and strong peaks at 1043.7 and 1114.5 cm^−1^ were due to the C–N and C–O stretching vibrations, respectively^95^.

In the FTIR spectrum of 28-homobrassinolide-Si-NPs, the broad and a strong peak at 1080.9 cm^−1^ was due to the S=O stretching vibrations and two simultaneous peaks at 648.6 and 998.9 cm^−1^ were caused by the weak O–Si–O and strong C=C bending vibrations, respectively^96^. In the FTIR spectrum of 28-homobrassinolide-Ca-NPs, a strong peak at 1628.8 cm^−1^ showed the stretching vibrations of C=O bond, and a pair of peaks at 1423.8 and 1483.5 cm^−1^ were due to the combined effects of stretching vibrations of strong Ca–O bond and strong bending vibrations of –OH groups. In addition, two individual peaks at 883.4 and 1110.7 cm^−1^ were due to the weak Ca–O bond and strong C–O stretching vibrations^97^. In the FTIR spectrum of 28-homobrassinolide-Zn-NPs, five individual peaks at 451, 667.2, 745.5, 872.2 and 1084.7 cm^−1^ were due to the strong ZnO bond, C=C bending vibrations and stretching and bending vibrations of ${{\text{SO}}}_{4}^{-2}$ ions^89^.

In the FTIR spectrum of ethephon, five sharp, weak and simultaneous peaks at 3000.5, 3213.0, 3309.9, 3406.8 and 3526.1 cm^−1^ indicated the strong stretching vibrations of –OH groups, due to the presence of intra-molecularly bonded alcoholic functional groups. In the next portion, two weak and broad peaks at 2703.3 and 2914.8 cm^−1^ were caused by the –OH and C–H stretching vibrations. Just before the fingerprint region of FTIR spectrum, the broad and strong peak at 1654.9 cm^−1^, and sharp and weak peak at 1751.8 cm^−1^ were caused by the stretching vibrations of carbonyl groups. In the next portion, two sharp and strong peaks at 1438.8 and 1498.4 cm^−1^ were mainly attributed to the C–H bending vibrations, due to the presence of methylene functional groups. In this series, some multiple peaks at 1222.6, 1271.0, 1312.0 and 1386.6 cm^−1^ were due to the stretching vibrations of P–O and P=O, indicating the presence of phosphate functional groups. Some additional pointed and sharp peaks at 820.0, 719.4 and 752.9 cm^−1^ were caused by the strong stretching vibrations of C–Cl bonds. Similarly, some multiple peaks at 674.6, 868.5 and 987.7 cm^−1^, and 1021.3, 1056.0 and 1110.7 cm^−1^ were due to the C=C and C–H bending vibrations and C–O stretching vibrations, respectively^98^. In the FTIR spectra of ethephon based nano-formulations, only characteristic peaks are described in detail and rest of the peaks are according to the FTIR spectrum of ethephon.

In the FTIR spectrum of ethephon-EDTA-NPs, two sharp and weak peaks at 3015.4 and 3526.1 cm^−1^, and two merged and broad peaks at 3313.6 and 3406.8 cm^−1^ were caused by the N–H and –OH stretching vibrations. In addition, a pointed, broad and strong peak at 1654.9 cm^−1^ and a sharp and shouldered peak at 7151.8 cm^−1^ were due to the C=O stretching vibrations^95^. In the FTIR spectrum of ethephon-Si-NPs, two pointed, sharp and weak peaks at 652.3 and 872.2 cm^−1^ were mainly attributed to the presence of O–Si–O and Ca–O bonds, respectively^96^. In the FTIR spectrum of ethephon-Ca-NPs, two broad and strong peaks at 1319.3 and 1565.5 cm^−1^ and a sharp, medium peak at 1722.0 cm^−1^ were due to the combined effects of stretching vibrations of P–O and P=O bonds and weak Ca–O bonds of reacting molecules, and stretching vibrations of C=C and C=O bonds, indicating the release of chlorine atoms from ethylene-releasing-compounds. Similarly, a pointed, sharp and independent peak at 872.2 cm^−1^ was mainly caused by the Ca–O bond stretching vibrations^97^. In the FTIR spectrum of ethephon-Zn-NPs, some pointed, sharp and singlet peaks at 451 and 652.3 cm^−1^ were due to the ZnO stretching vibrations and fundamental vibrations of ${{\text{SO}}}_{4}^{-2}$ ions. In the next portion, some multiple peaks at 1121.9 and 1155.5 cm^−1^ and 1358.8 and 1408.9 cm^−1^ were due to the fundamental vibrations of ${{\text{SO}}}_{4}^{-2}$ ions and strong S=O stretching vibrations, respectively^89^.

#### Laser induced breakdown spectroscopy (LIBS)

**Figure 3:**
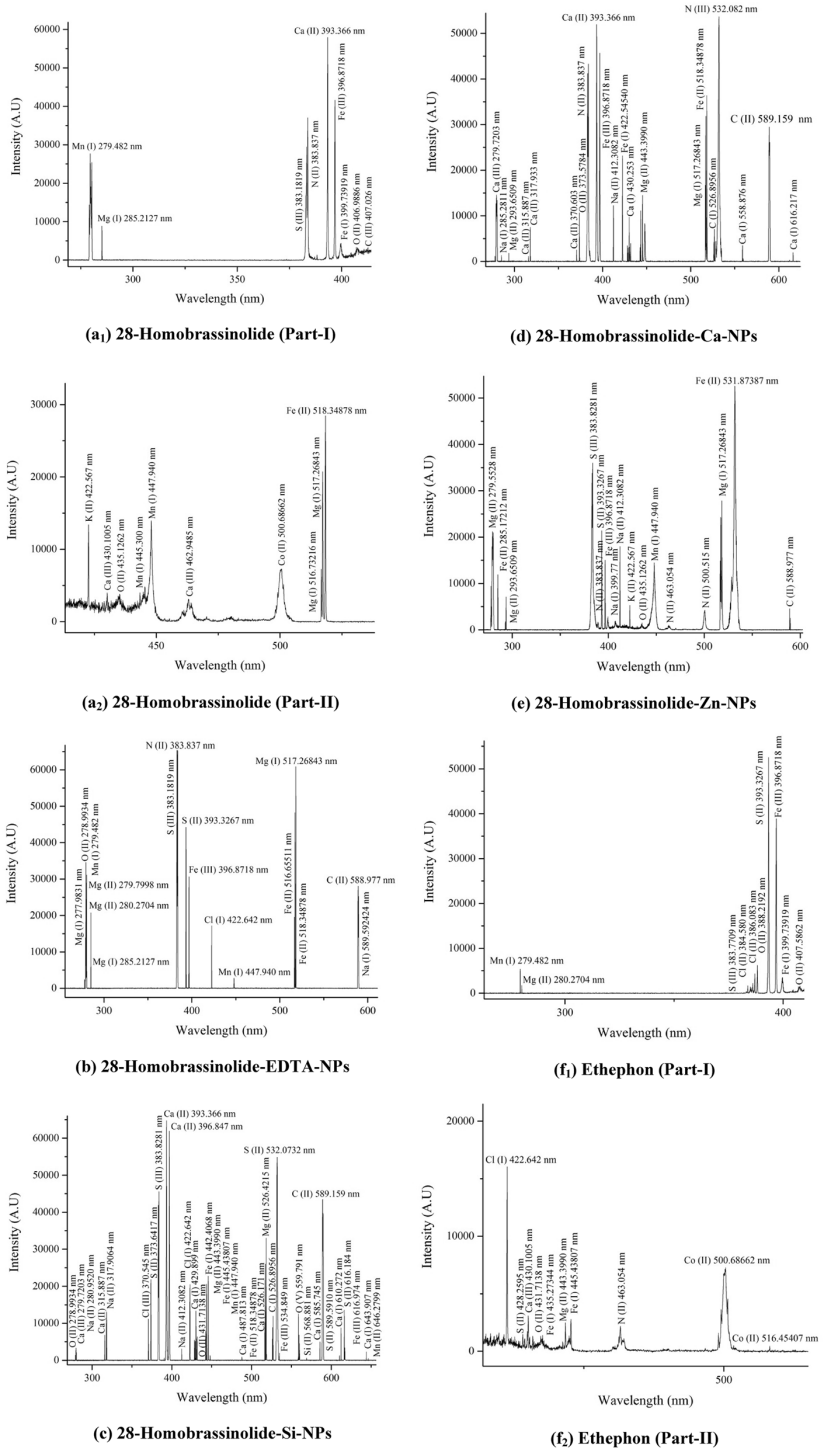

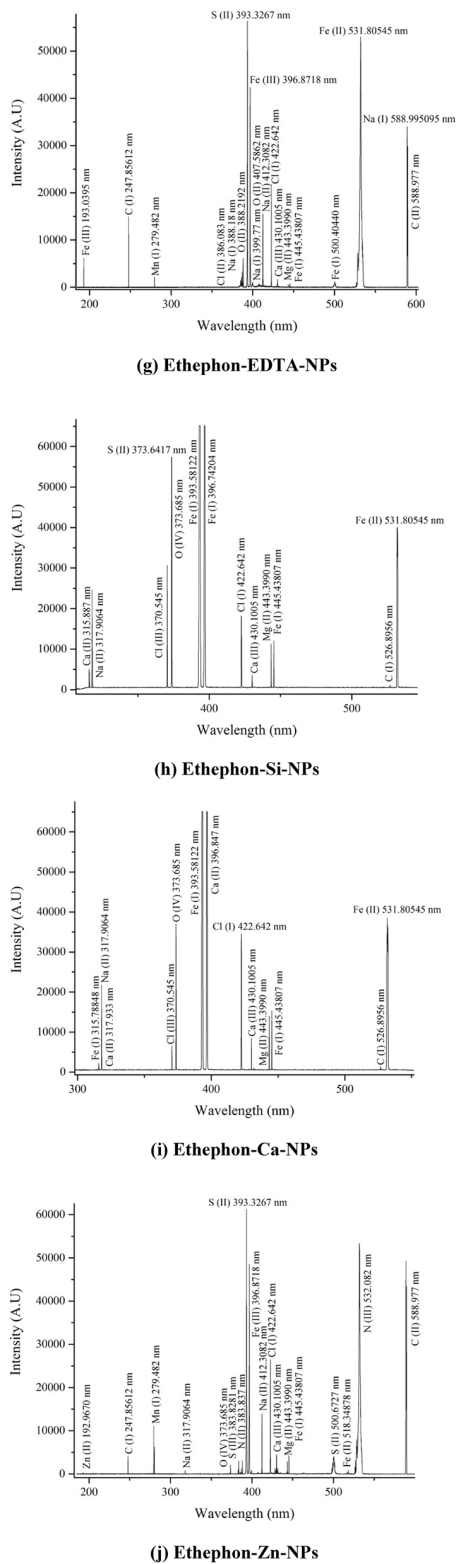
Laser induced breakdown spectroscopic (LIBS) analysis. EDTA-NPs: ethylene diammine tetraacetic acid based nanoparticles, Si-NPs: silica based nanoparticles, Ca-NPs: Calcium based nanoparticles and Zn-NPs: zinc based nanoparticles.

The LIBS spectra of 28-homobrassinolide, ethephon and their corresponding NPs are shown (Fig. 3). The elemental analysis of all above mentioned samples was made possible by obtaining the emitted wavelengths of analytical samples during the atomic transitions. These transitions were recognized by the use of NIST (National Institute of Standard and Technology) data base. Some of the major and minor identified elements were as follows: (a) 28-homobra-ssinolide: C(III) and O(II) (b) ethephon: Cl(I), O(II) and Cl(II) (c) 28-homobrassinolide-EDTA-NPs: Na(I), C(II), Cl(I), N(II) and O(II) (d) ethephon-EDTA-NPs: C(II), Na(II), O(II), Na(I), Cl(II) and C(I) (e) 28-homobrassinolide-Si-NPs: C(II), Ca(I), Si(II), O(V), C(I), Ca(I), Cl(I), S(III), S(II), Cl(III), Ca(II), Na(II), Ca(III) and O(II) (f) ethephon-Si-NPs: C(I), Ca(III), Cl(I), O(IV), S(II), Cl(III), Na(II) and Ca(II) (g) 28-homobrassinolide-Ca-NPs: C(II), N(III), C(I), Ca(I), Na(II), N(II), O(II), Ca(II), Na(I) and Ca(III) (h) ethephon-Ca-NPs: C(I), Ca(III), Cl(I), O(IV), Cl(III), Ca(II) and Na(II) (i) 28-homobrassinolide-Zn-NPs: C(II), O(II), Na(II), Na(I), S(II), N(II) and S(III) and (j) ethephon-Zn-NPs: C(II), N(III), Ca(III), Cl(I), S(II), N(II), S(III), O(IV), Na(II), C(I) and Zn(II)^99^.

#### Differential scanning colorimetry-thermogravimetric analysis (DSC-TGA)

**Figure 4:**
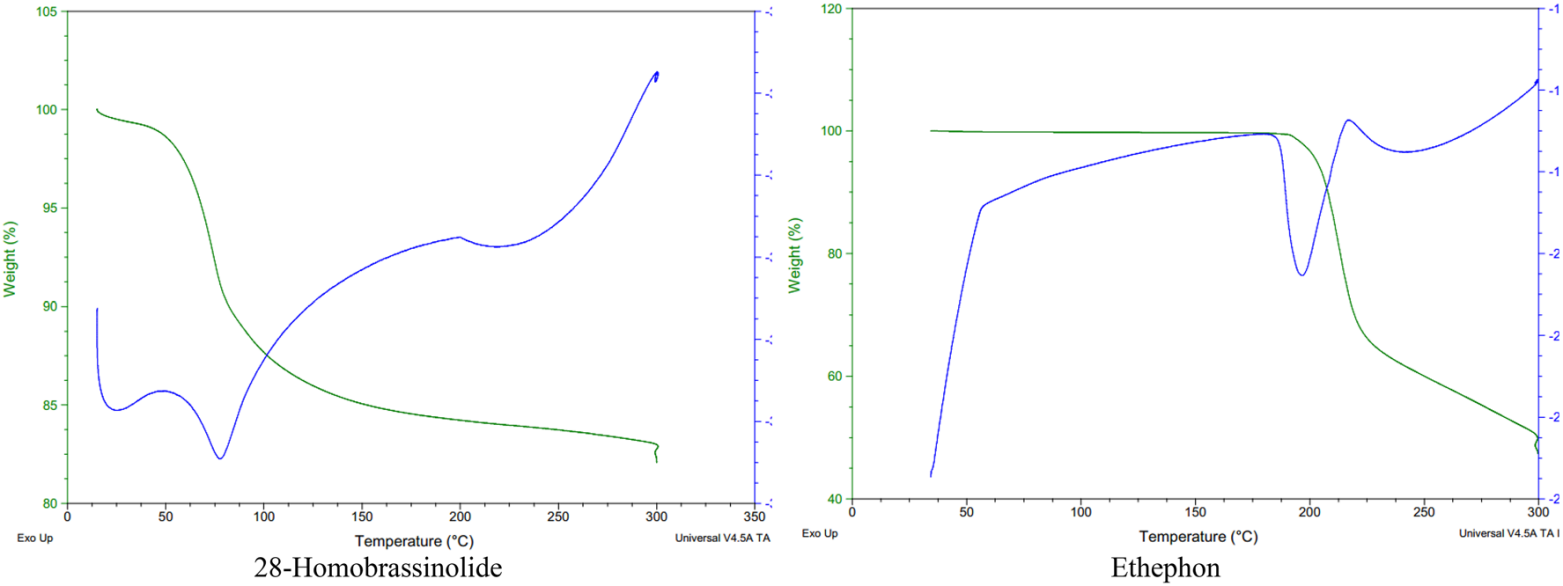
Differential scanning colorimetry–thermogravimetric analysis (DSC-TGA).

The DSC-TGA curves of 28-homobrassinolide and ethephon are shown (Fig. 4). The thermal analyses were performed to check the range of degradation temperature of 28-homo-brassinolide and ethephon, prior to the preparation of nano-structured PGRs. The DSC-TGA curves were obtained by heating the PGRs upto 300 °C, to check the gradual weight loss with respect to temperature. The thermogram obtained by the 28-homobrassinolide showed no weight loss till 50 °C, only 15% weight loss till 150 °C and again no weight loss up-till 300 °C. At the temperature greater than 300 °C, the exothermic reaction of thermal pyrolysis dominated and lead to the sudden weight loss upto 92 to 98%, as reported for brassinosteroid-modified polyethylene glycol micelles and various other structural analogues^100,101^. In case of DSC-TGA curves of ethephon, the thermogram showed no change in the weight till 200 °C, only 35% weight loss till 230 °C and almost 20% weight loss till 300 °C. In a previous study^102^, researchers reported the melting of ethephon at 73.3 °C and thermal decomposition upto 250 to 400 °C.

#### Scanning electron microscopy (SEM)

**Figure 5:**
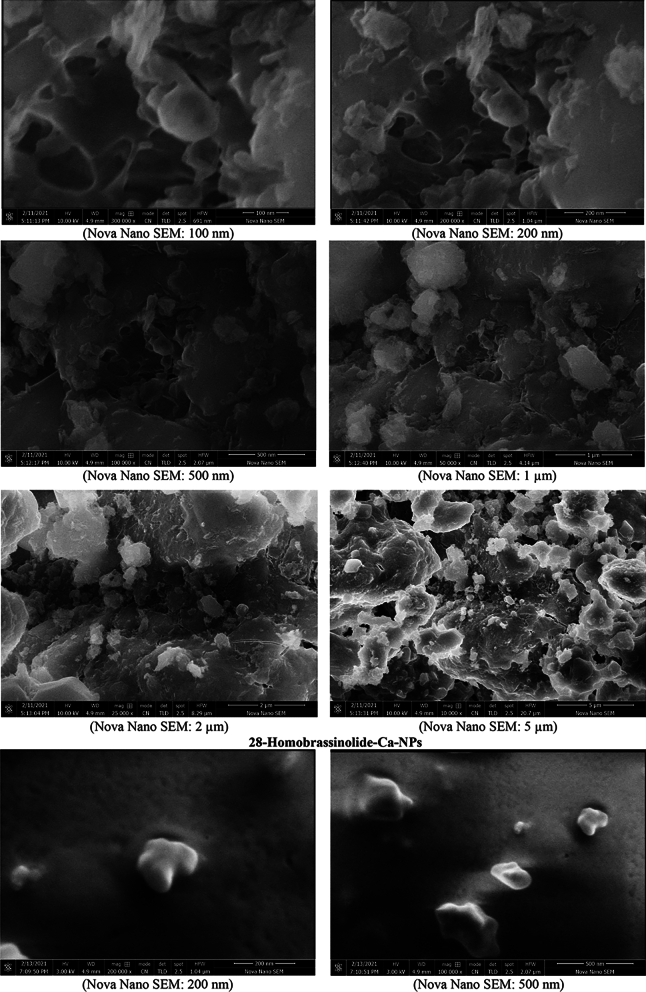

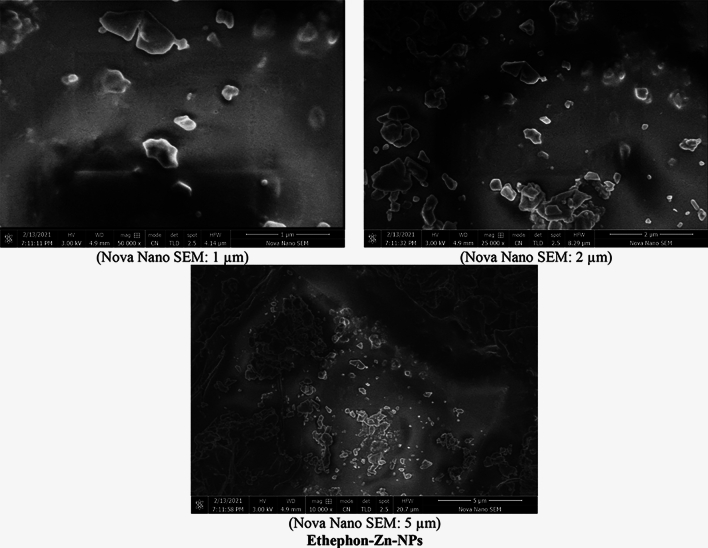
Scanning electron microscopic (SEM) images.

The SEM images of 28-homobrassinolide-Ca-NPs and ethephon-Zn-NPs are shown (Fig. 5). The SEM images of 28-homobrassinolide-Ca-NPs showed that the average particle size of prepared formulations ranged from 100 nm to 5 µm, having ovoid to spherical and irregular shape. The black spots and empty spaces in the SEM images are representative of the higher surface area to volume ratio, indicating the better assimilation of nano-structured PGR in the roots of *Mentha arvensis* L. These types of nano-formulations have not been reported yet, however literature strongly supports the obtained results and surface morphologies, with respect to the calcium based nano-fertilizers. The edgy structure, hollow areas, dark spots, and smaller particle size are some of the major characteristic features, responsible for the slow release of attached water molecules. These types of nano-formulations have proved to be ideal candidate for geographical areas, having restricted availability of water. In some recently published research articles^103–105^, researchers reported the significant improvements in surface properties and water holding capacities of nano-particles, as compared to their bulk counterparts. In another previous research article^106^, scientists also reported the larger surface area and improved characteristic properties of calcium oxide nano-particles due to the higher rate of agglomeration.

The SEM images of ethephon-Zn-NPs showed that the nano-particles ranged from 100 nm to 5 µm, having hexagonal to columnar and cuboidal to diamond like shape. The small particle size and large empty spaces are the major characteristic features of zinc based nano-formulations^107^. In a recently published research article^108^, researchers reported the spherical shape carbon balls enclosing the zinc sulphate nano-hybrids, for the fast and efficient delivery of zinc in the *Oryza sativa* L. In another research^109^, researchers reported the spherical shape of ZnO nano-particles, as a remedy for the rice crops. In a recent research article^110^, workers also used the zinc sulphate (ZnSO_4_) nano-particles on coffee (*Coffea arabica* L.). In a previous research work^111^, researchers reported the spherical and merged balls of zinc oxide nano-particles, prepared via microwave assisted method. In another article^112^, scientists mentioned the spherical aggregated ZnO nano-particles having particle size of 1 µm, prepared through green synthesis using *Hibiscus rosa-sinensis*.

#### Atomic absorption spectroscopy (AAS)

The zinc sulphate is a micro-nutrient of plants, having lower concentration (0.00022 g/L) as compared to the various other macro-nutrients and complexing agents. During the elemental analysis via LIBS, zinc was found to be below the detectable range of AvaSpec spectrometer. Therefore, the obtained concentrations of zinc were detected by AAS. The results showed that obtained concentrations of zinc in 28-homobrassinolides-Zn-NPs and ethephon-Zn-NPs were 0.125 ± 0.07 and 0.141 ± 0.14 ppm, respectively. These results were comparable with the added concentrations of zinc as per the previously published research article^55^. The slight variations in the final concentrations of zinc might be attributed to the leaching of Zn-NPs, during the process of washing and filtration.

#### Zeta potential and zeta size analysis

**Figure 6:**
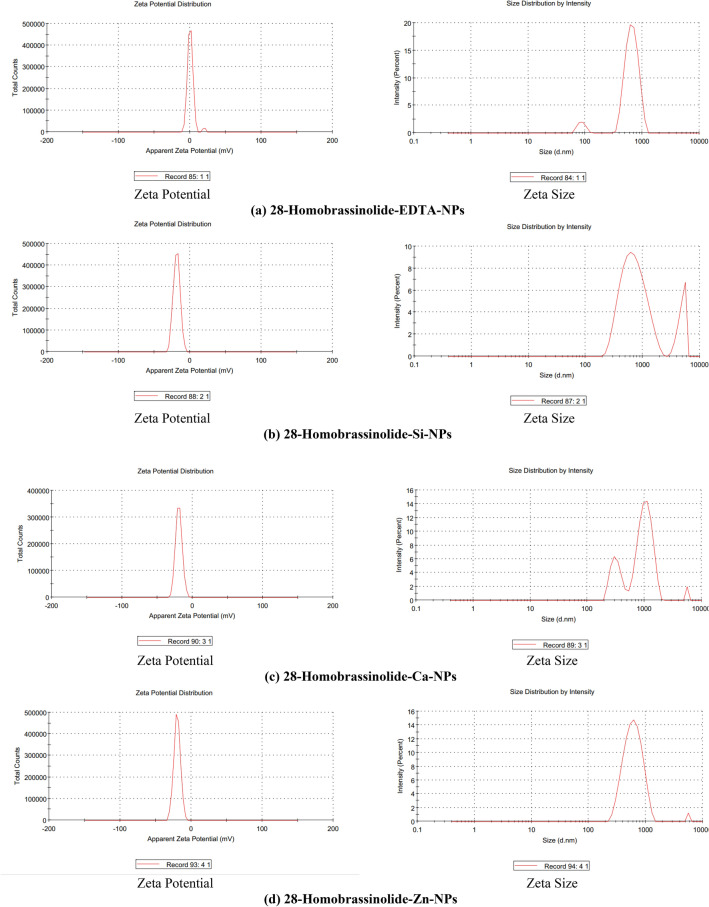

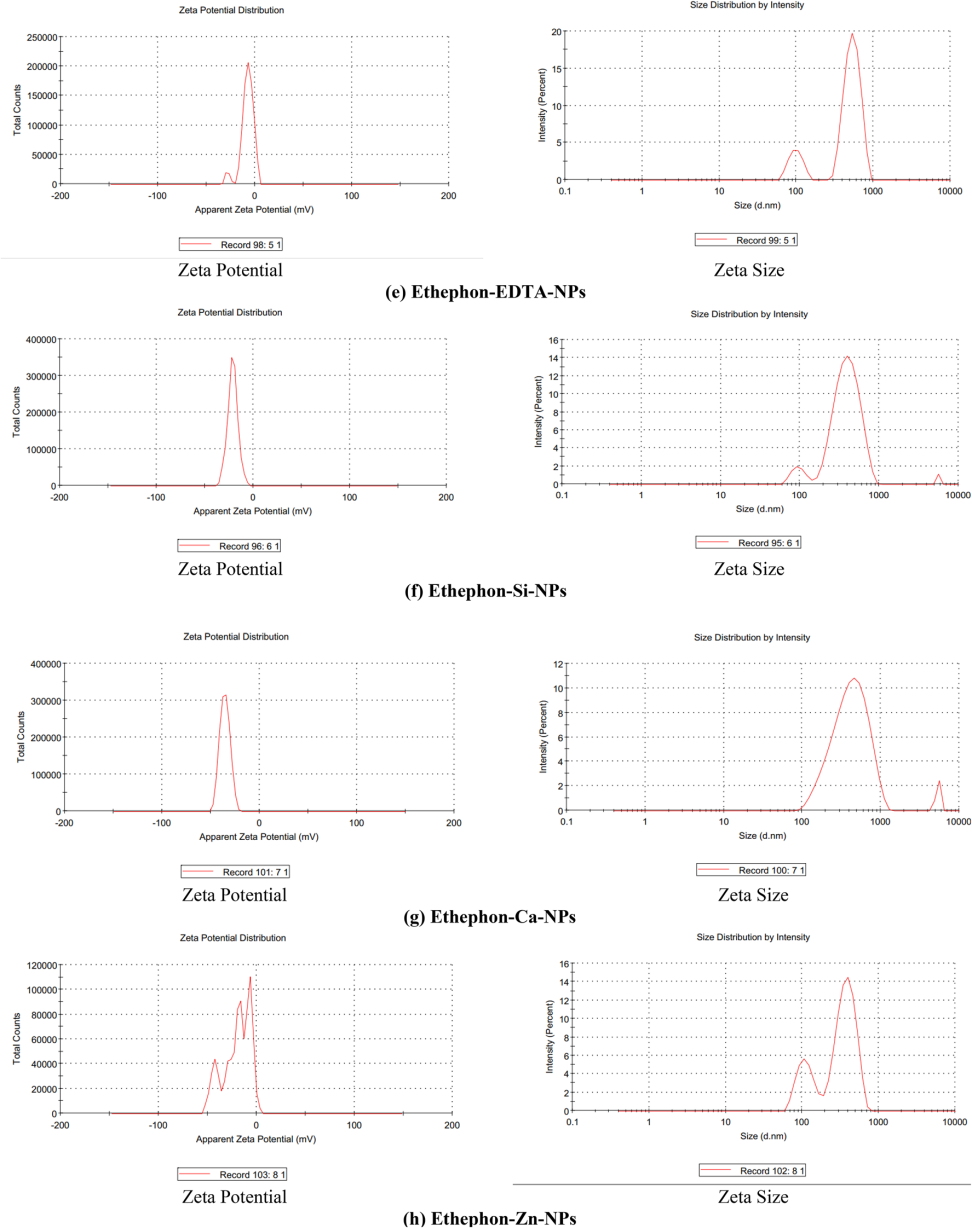
Zeta potential and zeta size analysis.

The visual outputs of zeta potential and zeta size analysis of nanostructured PGRs (28-homobrassinolide-EDTA-NPs, 28-homobrassinolide-Si-NPs, 28-homobrassinolide-Ca-NPs, 28-homobrassinolide-Zn-NPs, ethephon-EDTA-NPs, ethephon-Si-NPs, ethephon-Ca-NPs and ethephon-Zn-NPs) are shown (Fig. 6). In case of 28-homobrassinolide-EDTA-NPs, the average particle size, polydispersity index and zeta potential were 790.4 nm, 0.552 and 0.761 ± 4.61 mV. The obtained results are comparable with the particle size range and zeta potential of previously studied EDTA NPs functionalized with magnetic iron oxide. The value of polydispersity index indicates the uniform charge distribution on the entire surface of sample^113^. In case of 28-homobrassinolide-Si-NPs, the average particle size, polydispersity index and zeta potential were 846.2 nm, 0.444 and − 18.5 ± 4.68 mV. In a previous study, a group of researchers^114^ reported the particle size of mesoporous silica to be ˃ 300 nm, with higher surface area to volume ratio. In case of 28-homobrassinolide-Ca-NPs, the average particle size, polydispersity index and zeta potential were 1142 nm, 0.577 and − 19.3 ± 4.63 mV. In a recent research article, researchers^115^ prepared the calcium hydroxide [Ca(OH)_2_] nanoparticles (CHNPs) by using the calcium nitrate dihydrate [Ca(NO_3_)_2_⋅2H_2_O] having average particle size 350 nm with the uniform surface.

In case of 28-homobrassinolide-Zn-NPs, the average particle size, polydispersity index and zeta potential were 783.1 nm, 0.551 and − 19.9 ± 4.63 mV. These results are comparable with the previous study dealing with the preparation of zinc based nanoparticles. In this research, ZnO NPs showed the average particle size 735 nm using water as a dispersing media^116^. In case of ethephon-EDTA-NPs, the average particle size, polydispersity index and zeta potential were 801.1 nm, 0.642 and − 7.85 ± 6.65 mV. In case of ethephon-Si-NPs, the average particle size, polydispersity index and zeta potential were 419.6 nm, 0.490 and − 21.1 ± 5.33 mV. In the previous study, scientists and researchers^117^ also used the EDTA-NPs as a capping or encapsulating agent and reported the average particle size to be 28–33 nm. In case of ethephon-Ca-NPs, the average particle size, polydispersity index and zeta potential were 430.7 nm, 0.431, − 35.5 ± 5.18 mV. In a recent study, researchers^118^ prepared the calcium based nanoformulations having average particle size of more than 320 nm. These nanoformulations were found to be effective for efficient drug delivery for the treatment of cancer due to higher surface area and adsorption capacity. In case of ethephon-Zn-NPs, the average particle size, polydispersity index and zeta potential were 459.8 nm, 0.684, − 19.9 ± 13.6 mV. In a previous study, researchers^119^ also prepared the aqueous colloidal suspension of zinc sulfide nanoparticles ranging from 200 to 800 nm with negative value of zeta potential.

### Statistical analysis

The tabular form of data obtained from statistical analysis is given in (Tables 3, 4). The highest menthol (%) of *Mentha arvensis* L., grown by application of 28-homobrassinolide-L-I (79.72%), 28-homobrassinolide-EDTA-NPs-L-I (79.58%), 28-homobrassinolide-Si-NPs-L-I (54.9%), 28-homobrassinolide-Ca-NPs-L-III (80.84%) and 28-homobrassinolide-Zn-NPs-L-III (72.58%), and ethephon-L-I (67.59%), ethephon-EDTA-NPs-L-III (68.06%), ethephon-Si-NPs-L-III (80.94%), ethephon-Ca-NPs-L-III (81.53%) and ethephon-Zn-NPs-L-II (81.93%) were statistically compared with control (67.19%) and blank (63.93%) treatments. The p-values obtained by the one way analysis of variance (ANOVA) were less than 0.05, indicating the higher statistical variability among all the obtained percentages. The post-hoc Tukey HSD test also indicated the significant variations among the different pairs of treatments, in all the possible combinations^49^.

**Table 3:** Statistical analysis of percentage menthol contents of *Mentha arvensis* L. grown by applying 28-homobrassinolide.

(a)								
Treatment →	A	B	C	D	E	F	G	
Input data →	79.72 79.71	79.58 79.57	54.9 54.89	80.84 80.83	72.58 72.57	67.19 67.18	63.93 63.92	

(b)								
Treatment	A	B	C	D	E	F	G	Total
N	2	2	2	2	2	2	2	14
${\mathrm{\Sigma x}}_{{\text{i}}}$	159.43	159.15	109.79	161.67	145.15	134.37	127.85	997.41
$\overline{{\text{x}} }$	79.72	79.58	54.90	80.84	72.58	67.19	63.93	71.24
${\mathrm{\Sigma x}}_{{\text{i}}}^{2}$	12,708.96	12,664.36	6026.92	13,068.59	10,534.26	9027.65	8172.81	72,203.56
${{\text{s}}}^{2}$	0.0001	0.0001	0.0000	0.0001	0.0001	0.0000	0.0000	88.04
${\text{ss}}$	0.0071	0.0071	0.0071	0.0071	0.0071	0.0071	0.0071	9.38
${{\text{SE}}}_{\overline{{\text{x}}} }$	0.0050	0.0050	0.0050	0.0050	0.0050	0.0050	0.0050	2.51

(c)								
Source	SS	${\text{vv}}$	MS	F-statistic	p-value			
Treatment	1144.5106	6	190.7518	3,815,035.3741	1.1102 × 10^–16^			
Error	0.0003	7	0.0000					
Total	1144.5109	13						

(d)								
Pairs of treatments	Tukey HSD							
	Q statistics	p-value	Inference					
A vs B	28.0000	0.0010053	**p < 0.01					
A vs C	4964.0001	0.0010053	**p < 0.01					
A vs D	224.0000	0.0010053	**p < 0.01					
A vs E	1428.0000	0.0010053	**p < 0.01					
A vs F	2506.0000	0.0010053	**p < 0.01					
A vs G	3158.0001	0.0010053	**p < 0.01					
B vs C	4936.0001	0.0010053	**p < 0.01					
B vs D	252.0000	0.0010053	**p < 0.01					
B vs E	1400.0000	0.0010053	**p < 0.01					
B vs F	2478.0000	0.0010053	**p < 0.01					
B vs G	3130.0001	0.0010053	**p < 0.01					
C vs D	5188.0001	0.0010053	**p < 0.01					
C vs E	3536.0001	0.0010053	**p < 0.01					
C vs F	2458.0000	0.0010053	**p < 0.01					
C vs G	1806.0000	0.0010053	**p < 0.01					
D vs E	1652.0000	0.0010053	**p < 0.01					
D vs F	2730.0000	0.0010053	**p < 0.01					
D vs G	3382.0001	0.0010053	**p < 0.01					
E vs F	1078.0000	0.0010053	**p < 0.01					
E vs G	1730.0000	0.0010053	**p < 0.01					
F vs G	652.0000	0.0010053	**p < 0.01

**Table 4:** Statistical analysis of percentage menthol contents of *Mentha arvensis* L. grown by applying ethephon.

(a)								
Treatment →	A	B	C	D	E	F	G	
Input data →	67.59 67.58	68.06 68.05	80.94 80.93	81.53 81.52	81.93 81.92	67.19 67.18	63.93 63.92	

(b)								
Treatment	A	B	C	D	E	F	G	Total
N	2	2	2	2	2	2	2	14
${\mathrm{\Sigma x}}_{{\text{i}}}$	135.17	136.11	161.87	163.05	163.85	134.37	127.85	1022.27
$\overline{{\text{x}} }$	67.59	68.06	80.94	81.53	81.93	67.19	63.93	73.02
${\mathrm{\Sigma x}}_{{\text{i}}}^{2}$	9135.46	9262.97	13,100.95	13,292.65	13,423.41	9027.65	8172.81	75,415.90
${{\text{s}}}^{2}$	0.0001	0.0001	0.0000	0.0001	0.0001	0.0000	0.0000	59.27
${\text{ss}}$	0.0071	0.0071	0.0071	0.0071	0.0071	0.0071	0.0071	7.70
${{\text{SE}}}_{\overline{{\text{x}}} }$	0.0050	0.0050	0.0050	0.0050	0.0050	0.0050	0.0050	2.06

(c)								
Source	SS	${\text{vv}}$	MS	F-statistic	p-value			
Treatment	770.4759	6	128.4127	2,568,253.2344	1.1102 × 10^–16^			
Error	0.0003	7	0.0000					
Total	770.4763	13						

(d)								
Pairs of treatments	Tukey HSD							
	Q statistics	p-value	Inference					
A vs B	94.0000	0.0010053	**p < 0.01					
A vs C	2670.0000	0.0010053	**p < 0.01					
A vs D	2788.0000	0.0010053	**p < 0.01					
A vs E	2868.0001	0.0010053	**p < 0.01					
A vs F	80.0000	0.0010053	**p < 0.01					
A vs G	732.0000	0.0010053	**p < 0.01					
B vs C	2576.0000	0.0010053	**p < 0.01					
B vs D	2694.0000	0.0010053	**p < 0.01					
B vs E	2774.0000	0.0010053	**p < 0.01					
B vs F	174.0000	0.0010053	**p < 0.01					
B vs G	826.0000	0.0010053	**p < 0.01					
C vs D	118.0000	0.0010053	**p < 0.01					
C vs E	198.0000	0.0010053	**p < 0.01					
C vs F	2750.0000	0.0010053	**p < 0.01					
C vs G	3402.0001	0.0010053	**p < 0.01					
D vs E	80.0000	0.0010053	**p < 0.01					
D vs F	2868.0001	0.0010053	**p < 0.01					
D vs G	3520.0001	0.0010053	**p < 0.01					
E vs F	2948.0001	0.0010053	**p < 0.01					
E vs G	3600.0001	0.0010053	**p < 0.01					
F vs G	652.0000	0.0010053	**p < 0.01					

## Conclusions

- Excessive input of basic plant hormones and agricultural chemicals not only increase the cost of entire cropping system but also cause the wastage of soil nutrients via leaching.
- Use of nano-scale fertillizers and PGRs ensure the targeted delivery, controlled release, effective absorption and minimum input of agrochemicals without wastage.
- In the present study, nano-structured PGRs of 28-homobrassinolide and ethephon based on EDTA-NPs, Si-NPs, Ca-NPs and Zn-NPs were prepared to check their effects on the essential oil contents and menthol percentage of *Mentha arvensis* L.
- The nanostructured PGRs such as 28-homobrassinolide and ethephon have proved to be effective biostimulants in enhancing the release of secondary metabolites and production of menthol in essential oil of *Mentha arvensis* L.
- The highest essential oil percentage of *Mentha arvensis* L. was obtained by applying 28-homobrassinolide-Zn-NPs-L-II and ethephon-Ca-NPs-L-III while the highest menthol contents were obtained by the application of 28-homobrassinolide-Ca-NPs-L-III and ethephon-Zn-NPs-L-II.
- The nanostructured PGRs prepared in this research work are cost effective, economical, environmentally benign, photo resistant, thermally stable and non-toxic in nature with the minimum leaching potential.
- These nanoformulations are ideal to be introduced for commercial scale implementation in agricultural land areas where growth potential of industrially and medicinally valuable aromatic crops can be increased upto great extent. This can have a pronounced effect on the total GDP of developing agricultural countries.
- In the present study, major focus was to check the effects of prepared nano-formulations only on the menthol contents of essential oil of *Mentha arvensis* L. In future studies, the effects of these nanoformulations can be checked on all the isomeric forms of menthol with the detailed mechanism and complete mode of actions.

## Data Availability

All data generated or analysed during this study are included in this published article.
